# Translation and validation of a Japanese version of the irritable bowel syndrome-quality of life measure (IBS-QOL-J)

**DOI:** 10.1186/1751-0759-1-6

**Published:** 2007-03-03

**Authors:** Motoyori Kanazawa, Douglas A Drossman, Masae Shinozaki, Yasuhiro Sagami, Yuka Endo, Olafur S Palsson, Michio Hongo, William E Whitehead, Shin Fukudo

**Affiliations:** 1 Department of Behavioral Medicine, Tohoku University Graduate School of Medicine, 2-1 Seiryo, Aoba, Sendai 980-8575, Japan; 2 Department of Psychosomatic Medicine, Tohoku University Hospital, 1-1 Seiryo, Aoba, Sendai 980-8574, Japan; 3 Department of Comprehensive Medicine, Tohoku University Hospital, 1-1 Seiryo, Aoba, Sendai 980-8574, Japan; 4 Center for Functional GI & Motility Disorders, the University of North Carolina at Chapel Hill, Chapel Hill, NC 27599, USA

## Abstract

**Aims:**

To compare quality of life (QOL) for patients with irritable bowel syndrome (IBS) between the U.S. and Japan, it is indispensable to develop common instruments. The IBS-QOL, which is widely used in Western countries, was translated into Japanese as there has been a lack of Japanese disease-specific QOL measures for IBS.

**Methods:**

The original 34 items of the IBS-QOL were translated from English into Japanese through two independent forward translations, resolution, back translation, and resolution of differences. Forty nine patients who had GI symptoms but did not have any organic diseases (including 30 IBS patients diagnosed by Rome II criteria) were recruited from Tohoku University Hospital in Sendai, Japan and completed a Japanese version of the IBS-QOL (IBS-QOL-J) concomitant with a Japanese version of the IBS severity index (IBSSI-J) twice within 7–14 days.

**Results:**

The IBS-QOL-J demonstrated high internal consistency (Cronbach's alpha; 0.96) and high reproducibility (intraclass correlation coefficient; 0.92, p < 0.001). Convergent analyses confirmed that the overall score of IBS-QOL-J was significantly correlated with overall severity of IBS symptoms on the IBSSI-J (r = -0.36, p = 0.01) and with the individual items on the IBSSI-J that assess interference with life in general (r = -0.47, p = 0.001) and dissatisfaction with bowel habits (r = -0.32, p < 0.05). Eight patients who reported continuous abdominal pain in the past 6 months had significantly lower scores in the IBS-QOL-J than those who did not (53.7 +- 12.7 vs. 73.6 +- 19.5, p < 0.01). Age, sex, education or marital status did not affect scores on the measure.

**Conclusion:**

The IBS-QOL-J is a reliable instrument to assess the disease-specific QOL for IBS. Considering cross-cultural comparison, this measure is likely to be a valuable tool to investigate the QOL in Japanese patients with IBS.

## Background

Irritable bowel syndrome (IBS) is a common gastrointestinal disorder and is often associated with psychological distress [1,2]. People with IBS have a significantly diminished quality of life (QOL) [3,4]. Even though a large percentage of subjects with IBS do not seek medical care (approximately 75%) [5], IBS is associated with significantly more absences from work and school and with impaired QOL [3,4]. The impact on QOL in patients with IBS is often underestimated by friends and family members, and even by the patient's doctors because they are not disabled in any obvious way and there is no apparent impact on life expectancy.

How much of a burden illness is on an individual's life depends on several factors. It is becoming recognized that assessment of QOL associated with a person's illness should be taken into account in order to understand the burden illness on several medical conditions. We have demonstrated that both IBS patients and IBS non-consulters report not only more severe gastrointestinal symptoms and psychological distress but also more impaired health-related QOL than people who do not have IBS, as has been reported in other countries [6]. In contrast to reports form the United States [7], IBS patients were not different from IBS non-consulters for physical and psychological QOL scores on the SF-36 [8].

Although generic instruments like the SF-36 offer the opportunity to compare the impacts of different conditions on health status [9,10], disease-specific measures are more sensitive than generic measures of QOL to the effects of illness and the impact of treatment [9]. Within the gastroenterology field, disease-specific quality-of-life measures have been developed primarily for inflammatory bowel disease [11].

Patrick and Drossman developed and validated a specific QOL measure, the IBS-QOL, to assess the impairment of QOL in IBS [12]. The instrument defines "perceived quality of life" according to a needs-based model that considers QOL as the degree to which all or most needs are met [13]. It reveals patients' predominant concerns with high degree of specificity and attribution to the bowel symptoms associated with the condition [12]. Furthermore, they confirmed that this instrument is responsive to treatment in a clinical population of patients with FBD [14]. This instrument has been translated into several languages (i.e. Dutch, Spanish, French, Chinese and Korean) and is widely used [15-21].

There have been no studies on the cross-cultural differences in IBS between the U.S. and Japan due to the lack of common disease-related instruments. To compare QOL in patients with IBS between these countries, it is indispensable to develop disease-specific QOL measurements like the IBS-QOL in Japanese. The aim of this study was to test the reliability and validity of a Japanese version of the IBS-QOL (IBS-QOL-J).

## Methods

Forty-nine consecutive patients (27 female and 22 male) who suffered with chronic or recurrent abdominal pain, abdominal discomfort and/or altered bowel habit in Tohoku University Hospital, Sendai, Japan were invited to participate. All the participants completed the IBS-QOL-J (see Additional file 1) concomitant with a Japanese version of the IBSSI (IBSSI-J) [22] twice between 7–14 days interval to confirm the test-retest reproducibility. The Japanese version of the Rome II modular questionnaire, in conjunction with medical evaluation to exclude alternative diagnosis, was used to diagnose IBS [22]. None of the participants had any organic GI diseases or any other severe physical and/or psychological disorders. Age, sex, marital status, education, race, number of visits to physicians due to GI symptoms, and existence of continuous or nearly continuous abdominal pain in the past 6 months were also investigated (Table 1). This study was approved by the Tohoku University Ethics Committee (No. 2004-061). All the participants gave written informed consent.

**Table 1:** Characteristics of the sample (n = 49)

Sex	
Male	22
Female	27
*Age *(yrs, mean ± SD)	38 ±15
*Race*	
Japanese	49
Others	0
*Marital status*	
Single living alone	10
Single living with a partner	14
Married	24
Divorced	1
*Education*	
8th grade or less	3
Some high school	1
High school graduate	19
Some college or technical school	8
College graduate	18
*Diagnosis by Rome II criteria*	
Irritable bowel syndrome (IBS)	30
*IBS subtypes of bowel habit*	
IBS with constipation (IBS-C)	6
IBS with diarrhea (IBS-D)	14
Mixed or unspecified IBS	10
Other functional bowel disorders (FBD)	19
*Classification of symptom severity by IBSSI*	
Mild	15
Moderate	22
Severe	12

IBSSI: IBS severity index. Data were expressed as number of subjects except for age.

The Rome II modular questionnaire (RMIIQ) has been used as a diagnostic instrument for functional GI disorders according to Rome II diagnostic criteria [23]. This instrument includes 4 key questions used to define IBS plus 11 supportive items that address bowel habits. IBS is defined by abdominal discomfort or pain that was present during at least 3 weeks in the last 3 months and that has two of three features: (1) Relieved with defecation; and/or (2) onset associated with a change in frequency of stool; and/or (3) onset associated with a change in form (appearance) of stool [22,23]. Subtypes of IBS were assessed by predominant stool pattern; hard or lumpy stools >25% and loose (mushy) or watery stools <25% of bowel movements as IBS with constipation (IBS-C), and loose (mushy) or watery stools >25% and hard or lumpy stools <25% of bowel movements as IBS with diarrhea (IBS-D) [24].

The IBS Severity Index (IBSSI) was developed and validated in the U.K. to assess the major GI symptoms in IBS [25]. This scoring system is simple and consists of only 5 items each with 100-point scale (0, none; 100, worst). These items are severity and duration of abdominal pain, severity of abdominal distension, dissatisfaction with bowel habits (bowel score) and interference with life in general (QOL). The maximum total score is 500 points [22,25]. Recently, these two instruments have been translated into Japanese and validated for Japanese patients with functional bowel disorders (FBD) by our research group [22].

The IBS-QOL consists of 34 items with 5-point response scales (0 to 4). The IBS-QOL is scored for 8 subscales; dysphoria (8 items), interference with activity (7 items), body image (4 items), health worry (3 items), food avoidance (3 items), social reaction (4 items), sexual concerns (2 items) and relationships (3 items) [12]. Higher values indicate better QOL after converting the raw score on the IBS-QOL into 0 to 100 points. The process for the validation study of the IBS-QOL-J was as follows: The original 34 items of the IBS-QOL were translated from English into Japanese through two independent forward translations (by M.K. and S.F.), resolution, back translation by a native speaker of English, and resolution of differences. As a result of the discussion among the translators and the authors of the original version (D.A.D. and W.E.W.), no obvious difference of contents was found between the original and the back-translated versions.

Cronbach's alpha was calculated to assess the internal consistency reliability. A high internal consistency suggests that the scale is measuring a single construct. Reproducibility was assessed by comparing the total IBS-QOL-J score at baseline to a second one later. Convergent and discriminant validity involve comparing logically related measures to see if they are correlated more strongly (convergent) or more weakly (discriminant). The overall score for the IBSSI-J and the scores for the 5 components at baseline were used to assess convergent validity of the IBS-QOL-J. Strengths of association as the test-retest reliability or the convergent/construct validity were tested by a simple regression analysis, Pearson's correlation coefficient. Multiple regression analysis was used to confirm the relationships among the IBS-QOL-J score, age, gender, education and marital status.

## Results

Characteristics of the participants are shown in Table 1. The mean age with standard deviation (SD) of participants was 38 +- 15 years (19–79 years old). Thirty patients were diagnosed as IBS by the Japanese version of Rome II modular questionnaire, and the rest of them were considered to have other FBD. Six patients diagnosed as IBS were constipation predominant (IBS-C), 14 were diarrhea predominant (IBS-D) and 10 were not classifiable as either. Overall score of the IBS-QOL-J demonstrated high reproducibility (intraclass correlation coefficient, 0.92, p < 0.001) and high internal consistency (Cronbach's alpha, 0.96). Each individual score on the IBS-QOL-J also showed high reproducibility and relatively high internal consistency (Table 2). Response to the individual items significantly correlated with overall score of the IBS-QOL-J except for Q5 from the body image domain and Q32 from the health worry domain (Table 3).

**Table 2:** Reliability and validity for the overall and individual scores on the IBS-QOL-J

**IBS-QOL**	**Score (mean ± SD) (Time 1)**	**Cronbach's alpha (Time 1)**	**Intraclass correlation coefficient (ICC)**
Overall scale	70.3 ± 19.9	0.96	0.92^a^
Subscales (number of items)			
*Dysphoria (8)*	61.5 ± 27.1	0.94	0.88^a^
*Interference with activity (7)*	66.4 ± 24.7	0.86	0.94^a^
*Body image (4)*	82.9 ± 18.0	0.56	0.90^a^
*Health worry (3)*	76.0 ± 18.6	0.48	0.81^a^
*Food avoidance (3)*	59.5 ± 28.2	0.83	0.92^a^
*Social reaction (4)*	78.2 ± 19.2	0.76	0.85^a^
*Sexual concerns (2)*	84.7 ± 19.6	0.61	0.80^a^
*Relationships (3)*	71.3 ± 25.6	0.74	0.87^a^

^a^*p *< 0.001; Pearson's correlation coefficient.

**Table 3:** Evaluation of the utility of individual items on the IBS-QOL-J

Domain	Item	Correlation	Domain	Item	Correlation
*Dysphoria*	Q1	-0.83**	*Health worry*	Q4	-0.56**
	Q6	-0.86**		Q15	-0.74**
	Q7	-0.82**		Q32	-0.19
	Q9	-0.76**	*Food avoidance*	Q11	-0.45**
	Q10	-0.75**		Q23	-0.55**
	Q13	-0.72**		Q28	-0.59**
	Q16	-0.80**	*Social reaction*	Q2	-0.60**
	Q30	-0.89**		Q14	-0.54**
*Interference with*	Q3	-0.60**		Q17	-0.60**
*activity*	Q18	-0.78**		Q34	-0.79**
	Q19	-0.60**	*Relationships*	Q8	-0.66**
	Q22	-0.70**		Q24	-0.70**
	Q27	-0.75**		Q33	-0.70**
	Q29	-0.73**	*Body image*	Q5	-0.10
	Q31	-0.53**		Q21	-0.76**
*Sexual*	Q12	-0.30*		Q25	-0.61**
	Q20	-0.47**		Q26	-0.68**

*p < 0.05, **p < 0.01; Pearson's correlation coefficient between individual items and overall score of the IBS-QOL-J.

Validity of the IBS-QOL-J was confirmed by the significant correlations with measures of disease severity. The overall score of IBS-QOL-J was significantly correlated with overall severity of IBS symptoms on the IBSSI-J (r = -0.36, p = 0.01) and with the individual questions on the IBSSI-J that assess interference with life in general (r = -0.47, p = 0.001) and dissatisfaction with bowel habits (r = -0.32, p = 0.03, Table 4). The correlations between the overall score of the IBS-QOL-J and individual scores of abdominal pain (severity and duration) were weaker and were not statistically significant. Abdominal distension (severity) on the IBSSI-J was correlated to quality of life was as measured by the IBS-QOL-J.

**Table 4:** Correlations between overall score of the IBS-QOL-J and overall and individual scores of the IBSSI-J

	**IBS-QOL-J**	
	
	Correlation	Significance
IBSSI-J		
Overall	-0.36	0.01
Abdominal pain (severity)	-0.21	N.S.
Abdominal pain (duration)	-0.23	N.S.
Abdominal distension	-0.00	N.S.
Bowel movement	-0.32	0.03
Quality of life	-0.47	0.001

**Figure 1:**
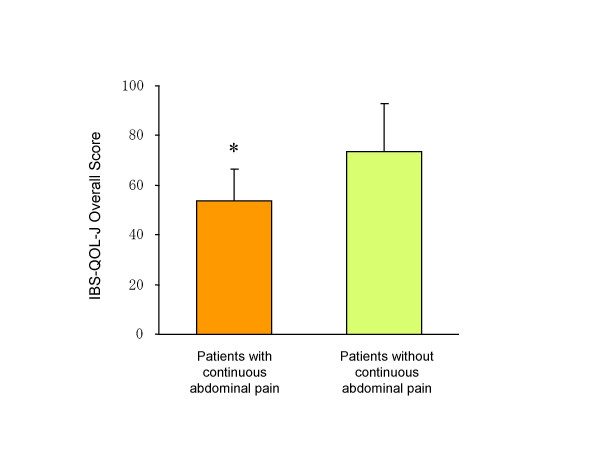
Comparison of the IBS-QOL-J scores between patients who reported continuous or nearly continuous abdominal pain and those who did not. Results were expressed as mean with standard deviation of the overall scores on the IBS-QOL-J. Patients who reported continuous abdominal pain showed a significant lower score than those who did not (*p < 0.01, Mann-Whitney U test).

Linear regression analyses revealed that the number of physician visits in the past 6 months was significantly associated with the overall score of IBS-QOL-J (r = -0.33, p < 0.05) but not with the overall severity score (r = 0.19, p > 0.1). Eight patients who reported continuous or nearly continuous abdominal pain in the past 6 months had significantly lower scores in the IBS-QOL-J than those who did not (53.7 +- 12.7 vs. 73.6 +- 19.5, p < 0.01, Fig. 1). There was no significant difference in the overall score or each of individual score on the IBS-QOL-J between patients who were diagnosed as IBS by the RIIMQ and those who were not (namely, the other FBD) in the participants (Table 5). Age, sex, education or marital status did not affect scores on the measure as a predictor variable. Most of the IBS-QOL-J scores in Japanese patients with IBS were quite comparable to those in the English version of the IBS-QOL in IBS patients in the U.S. There was no significant difference in the overall or each individual score on the IBS-QOL-J between subtypes of bowel movement in 30 patients diagnosed as IBS.

**Table 5:** Comparison of the IBS-QOL between Japan and the U.S

	*Score (mean ± SD)*		
	
	**IBS **(Japan)	**Other FBD **(Japan)	IBS* (U.S.)
Number	30	19	155
Female (%)	18 (60)	9 (47)	138 (89)
Age (yrs)	39 ± 17	37 ± 12	39 ± 12
IBSSI (Overall)	250 ± 95.5^a^	172 ± 89.8	- ^b^
IBS-QOL			
Overall	68.2 ± 19.7	73.6 ± 20.2	63.2 ± 18.5
Dysphoria	61.0 ± 26.8	62.2 ± 28.4	63.1 ± 23.9
Interference with activity	62.3 ± 25.8	72.9 ± 21.9	63.1 ± 22.3
Body image	80.0 ± 18.0	87.5 ± 17.4	62.5 ± 24.3
Health worry	73.1 ± 19.4	80.7 ± 16.7	59.2 ± 24.6
Food avoidance	55.3 ± 27.2	66.2 ± 29.1	43.4 ± 26.7
Social reaction	77.1 ± 18.9	79.9 ± 21.4	69.4 ± 22.9
Sexual concerns	89.2 ± 22.9	87.5 ± 13.2	73.5 ± 27.6
Relationships	72.2 ± 23.0	67.7 ± 29.8	72.3 ± 21.7

*IBS was diagnosed by Rome I criteria, data from Patrick, et al, 1998 (with permission, ref. 12). ^a^*p *< 0.01 vs other FBD. ^b^IBSSI was not investigated. Higher values indicate better QOL score for the IBS-QOL.

## Discussion

The Japanese version of the IBS-QOL (IBS-QOL-J) instrument was confirmed to be reliable and valid. This disease-specific QOL measurement shows high internal consistency for the overall score. The reproducibility over the two-week study period was excellent. The original version of the IBS-QOL has previously been shown to have high internal consistency and reproducibility [12]. On this original instrument, discriminant and convergent validity [12] and responsiveness [14] were also demonstrated. Not only severity of symptoms but also psychological well-being predicted this score [26]. Although Cronbach's alpha scores for most of the individual domains also resulted high (Table 2), the factor analysis revealed that a couple of individual items affected rather low alpha scores for the specific domains (Table 3). Further larger studies should be needed to confirm whether cross-cultural differences in patients' concerns might be associated with the inconsistencies in the present study.

Disease-specific quality of life instruments are sensitive and responsive to measuring treatment response over time; thus, they are especially useful in clinical research trials in which health status is analyzed [9]. Several disease-specific QOL measures for IBS or functional GI disorders have been developed (e.g. IBSQOL developed by Hahn et al. [27]). However, in most instruments, their responsiveness has not been demonstrated except for the IBS-QOL. In a systematic review by Bijkerk et al, it was shown that the IBS-QOL is the best of the five IBS-specific QOL scores to establish changes in health-related QOL [28]. On the other hands, the IBS severity index (IBSSI) is considered to be the best choice for a detailed IBS symptom assessment [28].

Recently, we have translated the Rome II modular questionnaire for IBS and the IBSSI into Japanese and have confirmed reliability and reproducibility in patients with functional bowel disorders (FBD) [22]. Our results in the present study show that the IBS-QOL-J is strongly correlated with the self-rating scales for the overall severity measure. Besides, patients who reported continuous or nearly continuous abdominal pain showed a lower overall score on the IBS-QOL-J than those who did not. On the other hand, our study did not confirm that there are significant differences in the QOL score among subtypes of bowel movement in patients with IBS. These results suggest that IBS patients who have abdominal pain continuously may have more impaired QOL despite predominant stool patterns. Furthermore, patients considered as frequent consulters according to a previous systemic review [29] show lower scores in the IBS-QOL-J. Previous reports on the original IBS-QOL show a significant association between the IBS-QOL and number of visit to physicians for IBS problems [14]. Thus, the findings of the present study are consistent with those of the original version of the disease-specific QOL measure for IBS.

The mean overall score of the IBS-QOL-J in patients with IBS was similar with that of original version [12] measured in the U.S. (68.2 vs. 63.2 points) despite different diagnostic criteria (Rome II vs. Rome I) and subject population (tertiary care vs. GI clinic plus advertisement). The mean overall score of the IBS-QOL-J in patients with IBS also showed a similar result with that measured by the Korean version of the IBS-QOL in South Korea [21]. Nevertheless, the mean individual scores of body image (80.0 vs. 62.5), health worry (73.1 vs. 59.2), food avoidance (55.3 vs. 43.4) and sexual concerns (89.2 vs. 73.5) on the Japanese version were over 10-point higher [12] (see Table 5).

Our results failed to confirm that the overall IBS-QOL-J score is significantly associated with the individual scores of the abdominal pain severity or pain duration in the IBSSI-J. We do not believe that gastrointestinal (GI) symptoms of the patients in this study were less severe because more than two-thirds demonstrated moderate to severe symptoms on the IBSSI-J according to the severity classification system on the original version [25] (see Table 1). Despite 41 of 49 patients had taken medical treatment for their GI symptoms including antispasmodic agents and antidepressants, they did not report lower abdominal pain scores compared with the rest of the patients who had not in the present study.

It has been demonstrated that the Japanese subjects are less prone to be accepting of pain behaviors [30] and express their sexual activities to someone [31] compared with people in the Western countries. There was no difference in the individual score of sexual concerns between married and unmarried patients in this study, in fact. When the sexual concerns are assessed in the Japanese patients, it should be taken into account that they may hesitate or avoid expression of such topics even if they have any sexual problems. Although we could not compare differences in these scores directly, cross-cultural difference between the countries (e.g. race, food, belief, social milieu and health-care system) might affect some dimensions of perception for the health-related QOL in patients with IBS.

The IBS-QOL-J appears to be a reliable instrument to assess the disease-specific QOL for IBS in Japanese patients. However, this validation study was cross-sectional, and thus could not investigate responsiveness. Moreover, our sample was relatively small and recruited from only the referred FBD patients. Further validation studies are warranted to investigate reliability and validity on the Japanese version of the IBS-QOL. It is important to assess not only severity of symptoms but also disease-specific QOL when considering the strategy for treatment for IBS since no biological measure is available for assessing IBS. Considering cross-cultural comparisons, these instruments are likely to be a valuable tool to investigate the QOL in Japanese patients with IBS.

## Supplementary Material

Additional File 1 Japanese version of irritable bowel syndrome quality of life (IBS-QOL-J) instrument. Click here for file
