# Supplementary material for: Translation and validation of a Japanese version of the irritable bowel syndrome-quality of life measure (IBS-QOL-J)
Source: Biopsychosoc Med. 2007 Mar 3;1:6. doi: 10.1186/1751-0759-1-6 (PMC1832201; doi:10.1186/1751-0759-1-6)
Supplement: Additional File 1 — Japanese version of irritable bowel syndrome quality of life (IBS-QOL-J) instrument. [file 1751-0759-1-6-S1.pdf]

Appendix

Japanese version of irritable bowel syndrome quality of life (IBS-QOL-J) instrument

Q1. おなかの症状のために、どうすることもできないように感じる
Q2. おなかの症状による臭いのために、恥ずかしい思いをしている
Q3. トイレに費やす時間の多さに悩んでいる
Q4. おなかの症状のために、他の病気に対する抵抗力が弱くなったと感じる
Q5. おなかの症状のために、体型が太ったと感じる
Q6. おなかの症状のために、日常生活をコントロールできなくなったと感じる
Q7. おなかの症状のために、日常生活をあまり楽しめなくなったと感じる
Q8. おなかの症状について話す時は、不愉快に感じる
Q9. おなかの症状について憂うつに感じる
Q10. おなかの症状のために、他人から孤立していると感じる
Q11. おなかの症状のために、食事の量に気をつけなければならない
Q12. おなかの症状のために、性的な行動に何らかの支障がある
Q13. おなかの症状があることに怒りを感じる
Q14. おなかの症状のために、他人に対するいら立ちを感じる
Q15. おなかの症状がだんだん悪くなっていくようで心配である
Q16. おなかの症状のために、過敏になっていると感じる
Q17. 私がおなかの症状のことを大げさに言うと人が思うのではないかと心配である
Q18. おなかの症状のために、物事があまり片づかないと感じる
Q19. おなかの症状のために、ストレス状況を避けなければならない
Q20. おなかの症状のために、性欲を失っている
Q21. おなかの症状のために、着るものや身につけるものが限られる
Q22. おなかの症状のために、一生懸命がんばるのを避けなければならない
Q23. おなかの症状のために、食事の内容に気をつけなければならない
Q24. おなかの症状のために、見知らぬ人といるのは困難である
Q25. おなかの症状のために、怠けていると感じる
Q26. おなかの症状のために、不潔に感じる
Q27. おなかの症状のために、長い旅行は難しい
Q28. おなかの症状のために、食べたいときに食べられないことに不満を感じる
Q29. おなかの症状のために、トイレの近くにいることが重要である
Q30. 私の生活はおなかの症状を中心に回っている
Q31. 排便が自分の思うようにならないことが心配である

Q32. 便を出せなくなることを恐れている
Q33. おなかの症状のために、身近な人達に迷惑をかけている
Q34. 誰も私のおなかの症状をわかってくれないと感じる

Subscale structure

憂うつ *Dysphoria*: items 1, 6, 7, 9, 10, 13, 16, and 30
活動制限 *Interference with activity*: items 3, 18, 19, 22, 27, 29, and 31
ボディー・イメージ *Body image*: items 5, 21, 25, and 26
健康に対する心配 *Health worry*: items 4, 15, and 32
食事回避 *Food avoidance*: items 11, 23, and 28
社会生活 *Social reaction*: items 2, 14, 17, and 34
性的問題 *Sexual*: items 12 and 20
人間関係 *Relationships*: items 8, 24, and 33

禁無断転載・使用

IBS-QOL 日本語版は著者の許可なしで使用することを禁じます. IBS-QOL 日本語版の著作権は、東北大学 金澤 素、福土 審に帰属します. 使用許可に関する連絡先 : 〒980-8575 仙台市青葉区星陵町 2-1 東北大学大学院医学系研究科行動医学 金澤 素、福土 審.

NOTE: The IBS-QOL must not be reproduced or utilized in any manner without prior approval of Novartis Pharmaceuticals Corporation and the authors. Requests for permission to utilize the IBS-QOL and to obtain a copy of the IBS-QOL User's Manual, contact either: Donald L. Patrick, Ph.D., M.S.P.H., Department of Health Services, H689, Box 357660, University of Washington, Seattle, Washington 98195-7660; or Douglas A. Drossman, M.D., University of North Carolina at Chapel Hill, CB# 7080, Room 726, Burnett Womack Building, Chapel Hill, North Carolina 27599-7080.
